# Associations between common mental disorders and menopause: cross-sectional analysis of the 2014 Adult Psychiatric Morbidity Survey

**DOI:** 10.1192/bjo.2023.82

**Published:** 2023-06-08

**Authors:** Amira Adji, Rebecca Rhead, Sally McManus, Natalie Shoham

**Affiliations:** Division of Psychiatry, University College London, UK; Department of Psychological Medicine, Institute of Psychiatry, Psychology and Neuroscience, King's College London, UK; Violence and Society Centre, City, University of London, UK; National Centre for Social Research, UK; Division of Psychiatry, University College London, UK; and Camden and Islington NHS Foundation Trust, St Pancras Hospital, UK

**Keywords:** Menopause, depression, generalised anxiety disorder, Adult Psychiatric Morbidity Survey, women's health

## Abstract

We investigated whether women who participated in a household survey in England were more likely to screen positive for possible generalised anxiety disorder and depression during and after menopause. We used logistic regression in secondary cross-sectional analyses of 1413 participants from the 2014 Adult Psychiatric Morbidity Survey data, adjusting for potential confounders (including age, deprivation score and chronic disease).

We found that participants who were post-menopausal were more likely to screen positive for possible depression compared with participants who were pre-menopausal (3.9% *v*. 1.7%; adjusted odds ratio 3.91, 95% CI 1.23–12.46), but there was no association with perimenopause. We found no evidence of an association between menopausal stage and possible generalised anxiety disorder or symptom score. Clinicians should be aware of the association between menopause and depression, to best support women. Future research could focus on to what extent associations are driven by somatic features, and how this might be modified.

Evidence suggests that depressive symptoms can increase among perimenopausal and post-menopausal women,^1–5^ although findings are mixed regarding clinical depression.^1^ Depressive symptoms have been found to associate with both hormonal levels and bothersome physical symptoms during perimenopause.^6,7^ Few studies have investigated the relationship between generalised anxiety disorder and menopause, and results have been inconclusive overall.^8^ Nevertheless, one study in China found an association between increased anxiety symptoms and both peri- and post-menopause,^9^ and a US study found that the perimenopausal period was associated with new-onset high anxiety levels.^10^ There have been few UK studies to compare depression or anxiety across different menopausal stages.

The aim of this study was to investigate the odds of screening positive for possible depression and generalised anxiety disorder (GAD) in women in the menopausal stages relative to the pre-menopausal stage. Our study builds upon previous work by using a nationally representative household survey. Our hypothesis was that women in the pre- and perimenopausal stages would have a higher chance of screening positive for both outcomes, and would report higher levels of anxiety and depression symptoms.

## Method

### Sample

We conducted a secondary analysis of data from the Adult Psychiatric Morbidity Survey (APMS) 2014.^11^ The APMS is a household survey conducted once every 7 years for the purpose of understanding the prevalence of mental health problems in England. Data was collected throughout 2014. The sampling process is described in detail elsewhere.^11^ We restricted analyses to participants aged 40–59 years who reported female gender and answered menopause-related questions (below).

### Exposure variable

Data was gathered by computer-assisted self-interviewing. Eligible participants were asked ‘Over the last 12 months, to what extent do you think you have been through or are going through the menopause?’ Participants who answered ‘Yes, I have been through menopause’ were defined as being in the post-menopause stage; ‘Yes, I am going through menopause’ as being in the perimenopause stage; and ‘No, not yet’ as being in the pre-menopause stage. We also combined the peri- and post-menopausal stages in separate analyses, for comparability with previous research.

### Outcome variables

Anxiety and depression symptoms were assessed in the face to face part of the interview, using the Clinical Interview Schedule–Revised (CIS–R).^12^ The interviewers used computer-assisted self-interviewing to ask if participants experienced symptoms in the past 7 days. Participants screened positive for possible depression or GAD if they met ICD-10^13^ criteria for moderate or severe depression or GAD.

We also used total CIS–R score after subtracting the scores for irritability, fatigue, somatic symptoms and sleep problems, which could be inherent symptoms of menopause.

### Covariates

We adjusted results for several covariates that could confound associations: continuous age in years; quintile of Index of Multiple Deprivation score;^14^ self-reported presence of chronic disease (asthma, cancer, epilepsy, diabetes and/or high blood pressure in the past 12 months);^15,16^ smoking status (none, ≤14 average daily number of cigarettes and ≥15 average daily number of cigarettes);^17,18^ Alcohol Use Disorders Identification Test score;^19–21^ and educational level (no qualifications, foreign/other, GCSE or equivalent, A level, vocational and degree).^19,22^

### Statistical analyses

We used logistic regression where the outcome was binary, and linear regression for continuous CIS–R score. All analyses were conducted in Stata version 17 for Mac and Windows.^23^ We used original APMS survey weighting, using the *svy* command in Stata, to account for selection and non-response bias. We report unweighted absolute numbers with weighted percentages. We conducted all analyses as complete-case analyses, unadjusted and adjusted for putative confounding variables.

## Results

Of the 7546 participants who were surveyed for the APMS 2014, 4488 reported female gender, 1510 were aged 40–59 years and 1413 (93.6% of those eligible) had answered all relevant menopause-related questions and constituted the analytic sample. Among these, 626 (45.2%) were classed as pre-menopausal; 409 (29.4%) were perimenopausal and 378 (25.5%) were post-menopausal. Further details can be seen in Table 1. The post-menopausal group tended to be older and have no qualifications compared with the other two groups.

**Table 1:** Characteristics of analytic sample according to menopausal stage

Characteristics, *N* = 1413	Menopause status, *n* (weighted %)			
	Pre-menopausal, *n* = 626 (45.2%)	Perimenopausal, *n* = 409 (29.4%)	Post-menopausal, *n* = 378 (25.5%)	Total
Age, years				
40–44	307 (49.9)	25 (6.1)	7 (1.4)	339 (24.7)
45–54	304 (47.6)	297 (71.2)	153 (41.1)	754 (52.8)
55–59	15 (2.6)	87 (22.8)	218 (57.5)	320 (22.5)
Screened positive for moderate to severe depression				
Present	16 (1.7)	14 (2.9)	20 (3.9)	50 (2.6)
Screened positive for generalised anxiety disorder				
Present	43 (5.9)	35 (7.6)	31 (6.6)	109 (6.6)
Quintile of Index of Multiple Deprivation score				
0.53–8.4	145 (22.4)	81 (19.2)	75 (18.9)	301 (20.6)
8.49–13.0	136 (21.7)	94 (23.9)	90 (26.3)	320 (23.5)
13.79–21	128 (20.2)	94 (22.2)	73 (19.7)	295 (20.6)
21.35–34	113 (17.9)	73 (18.0)	76 (20.0)	262 (18.4)
34.17–87	104 (17.7)	67 (16.8)	64 (15.2)	235 (16.8)
Education level				
No qualifications/other	74 (13.8)	46 (12.3)	79 (21.5)	199 (15.3)
GCSE or equivalent	164 (27.0)	138 (34.9)	123 (32.6)	425 (30.7)
A level	109 (16.2)	71 (16.7)	52 (14.4)	232 (15.9)
Vocational	51 (7.3)	45 (11.6)	27 (7.7)	123 (8.7)
Degree	219 (35.8)	106 (24.6)	90 (23.7)	415 (29.4)
Smoking				
None	287 (46.7)	166 (41.7)	147 (40.7)	600 (43.7)
≤14 average daily number of cigarettes	299 (47.5)	209 (49.1)	200 (52.4)	708 (49.2)
≥15 average daily number of cigarettes	40 (5.8)	34 (9.3)	31 (6.9)	105 (7.1)
Chronic disease				
Present	116 (19.2)	116 (27.6)	108 (28.8)	340 (24.1)
AUDIT score, median (interquartile range)	3 (1–6)	3 (1–6)	3 (1–5)	3 (1–6)

AUDIT, Alcohol Use Disorders Identification Test.

### Association between menopause stage and depression

Participants who were post-menopausal were more likely to screen positive for moderate to severe depression (3.9%) than those who were pre-menopausal (1.7%; odds ratio 2.31, 95% CI 1.10–4.89; *P* = 0.028) (Supplementary Table 1 available at https://doi.org/10.1192/bjo.2023.82). Notably, the prevalence in women who were post-menopausal was similar to the overall rate in women in the 2014 APMS (3.7%), and higher than that for men (2.9%).^24^ Following adjustment, both the size of the effect estimate and the statistical evidence of the association were increased (adjusted odds ratio 3.91, 95% CI 1.23–12.46; *P* = 0.027) because of negative confounding by age and alcohol dependence score. We found no statistically significant association between perimenopause and screening positive for depression before (odds ratio 1.69, 95% CI 0.73–3.92; *P* = 0.222) or after adjustment (adjusted odds ratio 2.17, 95% CI 0.89–5.28; *P* = 0.089). When combining peri- and post-menopausal groups, evidence of an association with possible depression persisted (before adjustment: odds ratio 1.98, 95% CI 1.00–3.90, *P* = 0.049; after adjustment: odds ratio 2.49, 95% CI 1.11–5.59, *P* = 0.027).

### Association between menopause stage and GAD

We found no statistical evidence of an association between menopause and screening positive for GAD, either before adjustment (perimenopausal group: odds ratio 1.33, 95% CI 0.78–2.27, *P* = 0.299; post-menopausal group: odds ratio 1.14, 95% CI 0.66–1.96, *P* = 0.640) or after adjustment (perimenopausal group: adjusted odds ratio 1.57, 95% CI 0.81–3.04, *P* = 0.182; post-menopausal group: adjusted odds ratio 1.43, 95% CI 0.64–3.21, *P* = 0.383) (Supplementary Table 1). There was also no evidence of association when menopausal groups were combined (before adjustment: odds ratio 1.24, 95% CI 0.78–1.96, *P* = 0.360; after adjustment: odds ratio 1.54, 95% CI 0.80–2.95, *P* = 0.195).

### Association between menopause and CIS–R scores

We found no statistical evidence for an association between total CIS–R score based on non-menopausal symptoms and menopause before adjustment (perimenopausal group: mean increase in score 0.46, 95% CI −0.28 to 1.19, *P* = 0.223; post-menopausal group: mean increase in score 0.55, 95% CI −0.15 to 1.25, *P* = 0.126) or after adjustment (perimenopausal group: mean increase in score 0.52, 95% CI −0.27 to 1.32, *P* = 0.197; post-menopausal group: mean increase in score 0.76, 95% CI −0.22 to 1.74, *P* = 0.129) (Supplementary Table 2). There was no evidence of association between CIS–R score and menopause when menopausal groups were combined (before adjustment: odds ratio 0.50, 95% CI –0.09 to 1.08, *P* = 0.094; after adjustment: odds ratio 0.59, 95% CI −0.17 to 1.34, *P* = 0.129).

## Discussion

### Main findings

Our results showed that women who are post-menopausal are more likely to screen positive for possible depression than women who are pre-menopausal. However, we found no significant association between perimenopause and possible depression, or between menopause and GAD, or total CIS–R score after accounting for features of menopause.

### Strengths and limitations

Strengths of this study included the use of a nationally representative sample and computer-assisted self-interviewing, which may have encouraged participants to be more honest in reporting sensitive topics.

A key limitation is cross-sectional design, which means that the temporal relationship between variables cannot be elucidated. It is possible that stress associated with depression and anxiety might influence the timing of menopause onset. The numbers who screened positive for GAD and depression were small, which reduced the power to detect clinically relevant associations and precluded further subgroup analysis (e.g. by ethnicity, younger age group or severity of symptoms). Results from the CIS–R were not validated by clinical interview, and this, alongside the inclusion of inherent features of menopause in the outcome measure for some of our analyses, could have led to false positives, or conversely false negatives if symptoms of depression were dismissed as symptoms of menopause. Menopause stage was also determined only by self-report, which could have led to some misclassification. We were unable to adjust for use of hormone replacement therapy, age at menopause onset or surgical menopause. Further research could make use of electronic health records to examine such objective measures, as well as mental health diagnoses and prescriptions.

### Interpretation

There is some evidence that rates of depression improve several years post-menopause, making our finding that post-menopause was the only period of elevated risk surprising.^22^ One possible reason that we did not find an association between menopause stage and GAD is that menopausal anxiety might differ from diagnostic criteria for GAD, and may have an irregular temporal pattern or unpredictable onset, meaning that it could be hard to detect in cross-sectional studies.^25^ Another possibility, given that we found no association with CIS–R score after removing somatic features of menopause, is that the apparent rise in common mental disorders in this phase is driven by inherent features of menopause, such as night sweats. Future longitudinal research could focus on to what extent this is true and how the association with depression might be modified. Healthcare professionals should be aware of the increased risk of depressive symptoms after menopause.

## Data Availability

The data that support the findings of this study are available from NHS Digital. Restrictions apply to the availability of these data, which were used under licence for this study.

## Supplementary material

For supplementary material accompanying this paper visit http://doi.org/10.1192/bjo.2023.82. click here to view supplementary material
