# Supplementary material for: Associations between common mental disorders and menopause: cross-sectional analysis of the 2014 Adult Psychiatric Morbidity Survey
Source: BJPsych Open. 2023 Jun 8;9(4):e103. doi: 10.1192/bjo.2023.82 (PMC10304854; doi:10.1192/bjo.2023.82)
Supplement: Supplementary file 1 [file S2056472423000820sup001.docx]

Supplementary Table 1

| Odds of Screening Positive for Depressive and Anxiety Subscales of CIS-R for Peri- and Post-Menopausal Women Relative to Premenopausal Women | | | | |
| --- | --- | --- | --- | --- |
| Characteristics (N = 1,413) | **OR [95% CI]** | ***p*** | **AOR [95%CI]** | **Adjusted *p*** |
| *Screening positive for moderate-severe depression* |  |  |  |  |
| Perimenopausal | 1.69 [0.73 - 3.92] | 0.222 | 2.17 [0.89 - 5.28] | 0.089 |
| Post-menopausal | 2.31 [1.10 - 4.89] | 0.028 | 3.91 [1.23 - 12.46] | 0.021 |
| Perimenopausal and post-menopausal combined | 1.98 [1.00 – 3.90] | 0.049 | 2.49 [1.11 – 5.59] | 0.027 |
| *Screening positive for GAD* |  |  |  |  |
| Perimenopausal | 1.33 [0.78 - 2.27] | 0.299 | 1.57 [0.81 - 3.04] | 0.182 |
| Post-menopausal | 1.14 [0.66 – 1.96] | 0.640 | 1.43 [0.64 - 3.21] | 0.383 |
| Perimenopausal and post-menopausal combined | 1.24 [0.78 – 1.96] | 0.360 | 1.54 [0.80 – 2.95] | 0.195 |

OR = Odds Ratio; AOR = Adjusted Odds Ratio (adjusted for age, chronic disease, deprivation score, education, and smoking and alcohol use).

Supplementary table 2

| Mean Difference in Scores for Common Mental Disorder Subscales (Without Core Features of Menopause) of CIS-R for Peri- and Post-Menopausal Women Relative to Premenopausal Women | | | | |
| --- | --- | --- | --- | --- |
| Characteristics (N = 1,413) | **Mean Difference [95% CI]** | ***p*** | **Adjusted Mean Difference [95%CI]** | **Adjusted *p*** |
| *CIS-R score (without core features of menopause)* |  |  |  |  |
| Perimenopausal | 0.46 [-0.28 - 1.19] | 0.223 | 0.52 [-0.27 - 1.32] | 0.197 |
| Post-menopausal | 0.55 [-0.15 - 1.25] | 0.126 | 0.76 [-0.22 - 1.74] | 0.129 |
| Perimenopausal and post-menopausal combined | 0.50 [-0.09 – 1.08] | 0.094 | 0.59 [-0.17 – 1.34] | 0.129 |

Adjusted = adjusted for age, chronic disease, deprivation score, education, and smoking and alcohol use
